# QuickStats

**Published:** 2013-10-25

**Authors:** Esther Hing, Amy Brown

**Figure:**
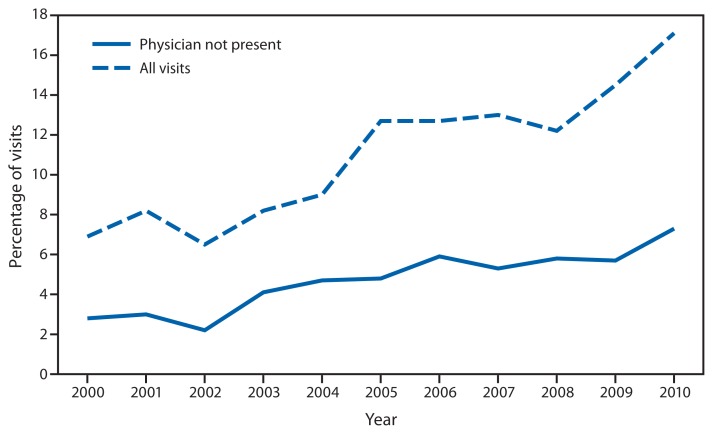
Percentage of Emergency Department (ED) Visits During Which a Patient Was Seen by a Physician Assistant or Nurse Practitioner, Overall and Without a Physician Present* — National Hospital Ambulatory Medical Care Survey, United States, 2000–2010 * Based on a national sample of visits to hospital EDs.

The percentage of hospital ED visits during which a patient was seen by a physician assistant or nurse practitioner increased from 7% in 2000 to 17% in 2010. The percentage of ED visits during which a patient was seen by a physician assistant or nurse practitioner and did not see a physician increased from 3% in 2000 to 7% in 2010.

**Source:** CDC. National Hospital Ambulatory Medical Care Survey. Available at http://www.cdc.gov/nchs/ahcd.htm.

